# Commentary: Interaction between facial expression and color

**DOI:** 10.3389/fnins.2017.00435

**Published:** 2017-07-28

**Authors:** Rocco Palumbo, Alberto Di Domenico

**Affiliations:** Università degli Studi “G. d'Annunzio” Chieti - PescaraPescara, Italy

**Keywords:** emotion, face recognition, emotion recognition, colors

Face expression recognition can be considered, among others, one of the most complex and developed perceptual skills (Haxby et al., 2000). Although this skill seems to play an important role in social communication (Johnson et al., 1991), its performance has been shown to change during the life-span (Mather and Carstensen, 2003; Di Domenico et al., 2016) and seems to be influenced by different variables such as clinical conditions (Sato et al., 2002; Altamura et al., 2016), culture (Jack et al., 2012), and facial features (Horry et al., 2015; Zebrowitz et al., 2015).

In the study, by Nakajima et al. (2017), the authors implemented two experiments where they investigated if face expression perception could be influenced by facial color and vice versa. In both experiments the authors used a set of faces created by morphing two facial expressions: fear to anger and sadness to happiness. One of the three color conditions: reddish-colored, neutral-colored, and bluish-colored was assigned to each face on the assumption that fear and sadness could be attributed to a bluish face, while anger and happiness to a reddish face (Nakajima et al., 2015). In the first experiment participants were asked to identify, as quickly and accurately as possible the facial expression while ignoring its facial color. The results of experiment 1 showed that facial color modulated the facial expression discrimination and the reaction times (RT) in the discrimination task. Specifically, reddish-colored faces enhanced anger perception, while bluish-colored faces enhanced sadness perception In the second experiment, instead, subjects were asked to identify the facial color, while ignoring its facial expression. The results of experiment 2 showed that facial color perception was significantly shifted only for the sad expression, suggesting a limited effect of facial expression on facial color perception. The results of these two experiments showed that subjects recognized the bluish-colored faces as sadder compared to the reddish- and neutral-colored ones and the reddish-colored faces as angrier than the bluish- and neutral-colored faces. Furthermore, based on the RT analysis the authors determined that the RT for reddish-colored faces were shorter compared to the natural-colored faces. All together these results brought the authors to hypothesize a link between facial color and emotion perception, supporting the existing literature that link facial color to emotion perception.

One interpretation that can be added to the findings of Nakajima et al. (2015) is that color could have enhanced the emotional content of the face stimuli by improving the performance on the recognition task that subjects were asked to perform. Emotions have been shown to improve the performance on cognitive tasks in different domains, such as memory, learning, and perception (Fairfield et al., 2013, 2015a; Mammarella et al., 2016b; Palumbo et al., 2017a,b). A well-known effect is the positive bias (Kennedy et al., 2004). It has been shown, for example, that older adults tend to prefer positive contents by remembering and recognizing better positive stimuli compared to younger adults, who seem to perform better on negative contents (Mather and Carstensen, 2003; Fairfield et al., 2015b; Mammarella et al., 2016c). In a study of Di Domenico et al. (2015), for example, it has been shown that, in a dynamic task of face expression recognition, RT are influenced by the emotional content of the facial expression suggesting a preference for emotional expressions compared to neutral expressions. Furthermore, recent studies that have investigated the influence of color on stimuli, have shown that color may carry an affective connotation able to influence emotional reactions of the participants (e.g., O'Connor, 2011; Mammarella et al., 2016a). The relation between a specific color and its evoked valence has been largely investigated suggesting that colors such as red are associated with negative emotions, while green seems to evoke positive emotions contrary to blue that is often associated with fear and sadness (e.g., Elliot and Aarts, 2011; Nakajima et al., 2015; Mammarella et al., 2016a). Taken all together, these findings can lead to the hypothesis that in the study of Nakajima et al. the color of the face could have enhanced the emotional valence of the stimuli facilitating the recognition task. Not only the authors supported the findings that attribute certain color to a specific emotion, but they also showed a reduction in the RT for reddish-colored faces on anger perception compared to natural-colored faces. Considering the sample of participants recruited by the researchers (younger adults; mean age = 23.30), a reduction in the RT for the reddish-colored faces seems to be in line with previous researches suggesting a preference for negative emotions in younger adults (Mather and Carstensen, 2003; Kennedy et al., 2004). To support the latest hypothesis, further investigations that would involve recruiting participants of different ages and races, is required. In fact, one of the variables that has not been considered by Nakajima et al., is whether the color of the faces would influence participants of different ages, or races, differently in the facial expression recognition task. Furthermore, it should be noted that the authors did not report whether they controlled or balanced the emotional intensity of the faces used in the study. In fact, stimuli of different emotional intensity may introduce a potential bias in the results, especially in the fear-to-anger pairs. Another factor that should be taken into consideration is whether or not clinical conditions of the participants, such as mood disorders like depression, may affect the results. According to previous studies (e.g., Leppänen, 2006), it has been demonstrated that participants with depression tend to interpret neutral faces as negative faces.

The study of Nakajima et al. (2017) showed how color can influence the facial expression recognition. This research, together with future studies can lead to a better understanding of how high-level perception is modulated and how variables such as physical features, emotions, and colors interact with each other to produce the final perception.

## Author contributions

All authors listed have made a substantial, direct and intellectual contribution to the work, and approved it for publication.

### Conflict of interest statement

The authors declare that the research was conducted in the absence of any commercial or financial relationships that could be construed as a potential conflict of interest.
